# Unilateral antibiotic-induced acute eosinophilic pneumonia on the operative side after surgery for primary lung cancer: a case report

**DOI:** 10.1186/s40792-020-00803-2

**Published:** 2020-02-19

**Authors:** Yasufumi Goda, Tsuyoshi Shoji, Hiromichi Katakura

**Affiliations:** Department of Thoracic Surgery, Otsu Red Cross Hospital, 1-1-35, Nagara, Otsu, Shiga 520-8511 Japan

**Keywords:** Acute eosinophilic pneumonia, Antibiotics, Postoperative pneumonia, Lung cancer

## Abstract

**Background:**

Acute eosinophilic pneumonia (AEP) is a rare idiopathic lung disease characterized by pulmonary eosinophilia. The epidemiology of AEP remains understudied; however, past reports have reported that AEP can be caused by an allergic reaction to medications, such as antibiotics or inhaled antigens, such as tobacco smoke. AEP usually occurs bilaterally. However, we encountered an unusual case of antibiotic-induced eosinophilic pneumonia showing unilateral consolidation just on the operative side, which was initially diagnosed as postoperative bacterial pneumonia and treated with antibiotic therapy. The prescribed antibiotics paradoxically provoked AEP and worsened the patient’s condition. Here, we report this antibiotic-induced AEP case showing unilateral consolidation only on the operative side which could be triggered by surgery for primary lung cancer.

**Case presentation:**

A 74-year-old man underwent right upper lobectomy for lung adenocarcinoma. On postoperative day (POD) 9, an interstitial shadow appeared in the right lower lung field of the chest radiographs, along with a fever of 38.5 °C, dyspnea needing oxygen supplementation, and increased purulent sputum production, suggesting postoperative bacterial pneumonia. Despite administration of the broad-spectrum antibiotic, meropenem, the fever did not improve, and pulmonary opacity gradually worsened. Blood analysis showed increased peripheral eosinophils at 1182/mm^3^. The meropenem treatment was discontinued and bronchoscopy was performed for further evaluation, and the bronchoalveolar lavage fluid assessment showed a remarkable increase in the eosinophil population (51%). The drug lymphocyte stimulation test (DLST) for meropenem was positive. We diagnosed the patient with antibiotic-induced unilateral AEP, after which corticosteroid treatment was initiated. The patient subsequently improved and the infiltration in the right lower lung field completely disappeared. The patient was discharged on POD 43 without oxygen supplementation and is doing well without tumor recurrence 16 months after the surgery.

**Conclusions:**

Unilateral drug-induced AEP is rare. Nonetheless, it should be recognized as a differential diagnosis of postoperative pneumonia even in cases of a unilateral radiographic infiltration, because the lung operation itself could trigger this type of AEP.

## Background

Acute eosinophilic pneumonia (AEP) is a rare disorder of heterogeneous diffuse parenchymal lung disease that is clinically distinct from chronic eosinophilic pneumonia and was first reported as an idiopathic disease by Allen et al. [1]. AEP is characterized by marked accumulation of infiltrating eosinophils in the alveolar space and interstitium. The epidemiology of AEP remains unknown and understudied; however, its pathological conditions can be caused by allergic reactions, secondary to medication and inhalation exposure [2]. AEP usually occurs bilaterally, and the most common features identifiable on a chest radiograph of patients with AEP are bilateral ground glass attenuation mixed with consolidation and bilateral pleural effusion in > 90% of the cases [3, 4].

For the above reasons, in unilateral AEP cases, reaching an accurate diagnosis is difficult.

Here, we report the case of unilateral antibiotic-induced AEP on the operative side which could be triggered by lung surgery for primary lung cancer.

## Case presentation

**Fig. 1:**
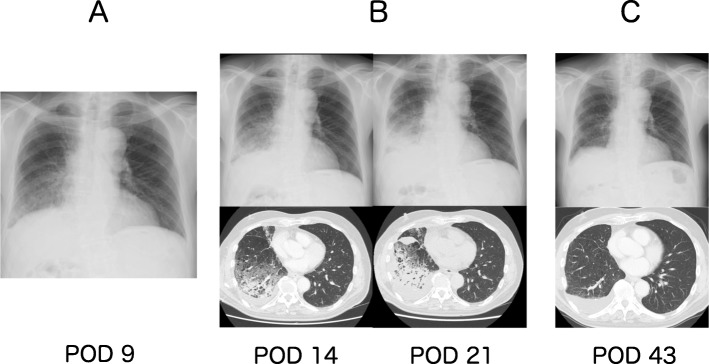
Image findings. **a** On POD 9, unilateral consolidation appeared on the right lower field. Bacterial pneumonia was thought to have occurred as a common cause of postoperative complication and broad antibiotic therapy using meropenem was started. **b** After the initiation of meropenem, on POD 14, chest X-ray radiographs and computed tomography scans showed gradually worsening (ground glass, reticular shadows, consolidation, and pleural effusion only on the right operative side). On POD 21, the consolidation on the right side had exacerbated regardless of the administration of meropenem. Thus, we started to question if meropenem itself provoked and worsened the patient’s condition by causing drug-induced AEP. **c** On POD 43, after cessation of meropenem and use of oral prednisolone, the abnormal shadow in the chest X-ray radiographs and computed tomography scans completely disappeared and the patient responded to treatment

**Fig. 2:**
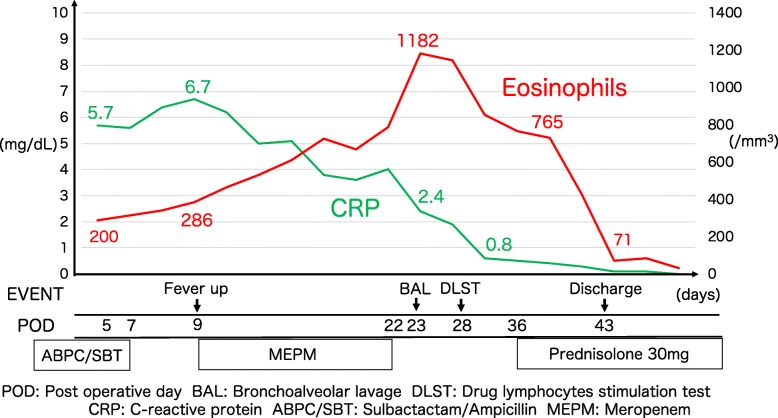
Clinical course. This figure shows the clinical course of the patient

A 74-year-old man underwent right upper lobectomy for lung cancer. The intraoperative findings showed that his lung was fragile because of severe emphysema, and minor air leakage was found near the staple line of the middle and lower lobes during the air leakage test. We used a polyglycolic acid sheet to cover the air leakage points and interlobar staple lines. Sulbactam/ampicillin was administered prophylactically after surgery until the chest drain was removed on postoperative day (POD) 7, due to complicated conditions with significant medical history: current smoker, hypertension, severe emphysema, aortic dissection, and diabetes mellitus. Chest radiographs on postoperative day 9 showed an interstitial shadow in the right lower lung field of the patient, who presented with a fever of 38.5 °C, dyspnea needing oxygen supplementation, and increased purulent sputum production containing gram-negative rods with phagocytes. The patient was administered the broad-spectrum antibiotic, meropenem, with the diagnosis of postoperative bacterial pneumonia resistant to sulbactactum/ampicillin for these clinical presentations. Despite treatment with meropenem, subsequent radiographs showed gradual worsening of the abnormal shadows of the right lower lung field (Fig. 1a, b). Laboratory analysis of the hemogram revealed gradual decreases in the values of the inflammatory parameters (white blood cells, from 10,500 to 7300/mm^3^; neutrophils, from 8001 to 4497/mm^3^, from 76.2 to 61.6%) but a gradual increase in eosinophil count (from 286 to 1182/mm^3^, from 4.2 to 16.2%; Fig. 2). After cessation of the meropenem therapy on POD 23, bronchoscopy was performed for further evaluation, and the bronchoalveolar lavage fluid (BALF) showed that the proportion of eosinophils was 51% (Table 1).

**Table 1:** BALF data. Results of the BALF cell analysis

Total cell count 3.0 × 10^5^ cells/ml	
Cell population (%)	
Eosinophils	51.0%
Histiocytes	30.0%
Lymphocytes	17.7%
Neutrophils	1.3%
Lymphocytes CD4/CD8	3.61

The drug lymphocyte stimulation test (DLST) was negative for sulbactam/ampicillin, but positive for meropenem. Based on the clinical course, the laboratory results of BALF, and positive tests in the DLST, we reached the correct diagnosis of drug-induced pneumonia caused by meropenem. The patient showed remarkable improvement with corticosteroid treatment, 30 mg of oral prednisolone as an initial dose. The dosage of prednisolone was gradually tapered down by 5 mg for 6 weeks after confirmation of steady conditions and was halted subsequently. After this treatment, the abnormal shadows on the radiographs remarkably disappeared (Fig. 1c) along with improvements in clinical conditions. The patient was discharged on POD 43 without oxygen supplementation. He is doing well without tumor recurrence 16 months after surgery with tegafur/uracil adjuvant chemotherapy.

## Discussion

We report the unilateral antibiotic-induced AEP case as a postoperative complication confused with prolonged postoperative bacterial pneumonia.

Considering the clinical course of the patient, we assume that the patient had bacterial pneumonia on the operative side on POD 9 and that meropenem was effective for bacterial pneumonia but simultaneously provoked antibiotic-induced AEP.

Clinical features such as acute fever, dyspnea, and malaise were non-specific, but peripheral blood eosinophilia at the initial presentation was a clue to diagnose antibiotic-induced AEP.

In this case, retrospectively, prolonged postoperative sulbactam/ampicillin administration until POD 7 should not have been done because prolonged antibiotic prophylaxis (> 48 h) is not recommended due to the increasing risk of resistant bacteria and *Clostridium difficile* infection [5]. This postoperative prophylactic administration resulted in confusion, leading to the difficulty of accurate diagnosis.

In recent years, tobacco smoking has been the most frequently reported trigger in causing AEP. Drug-induced AEP has been also reported. Antibiotics, nonsteroidal anti-inflammatory drugs (NSAIDs), and serotonin reuptake inhibitors are commonly associated with AEP [6]..

The pathogenesis of AEP is not fully known, however, is related to an acute type I hypersensitivity reaction triggered by offending agents such as cigarette smoke or drugs.

To respond to the offending agents, a cascade of immune events occur leading to the generation of inflammatory cytokines that promote an accumulation and activation of eosinophils in the lung parenchyma. Thus, inflammatory cytokines play an important role in the formation of AEP [7].

On the other hand, surgical stress activates the immune cells followed by the release of various inflammatory cytokines in response to whole body stress and to maintain homeostasis [8]. We performed video-assisted right upper lobectomy with general anesthesia, which caused stress damage to not only the whole body but also the remaining right middle and lower lobes which were handled from side to side during the video-assisted thoracic surgery (VATS) for lobectomy to obtain the surgical field of view.

We assume the reason why the unilateral consolidation appeared just in the remaining lobes was mainly due to the change of cytokine balance by the stimulation of lung operation.

In fact, past reviews reported that the same postoperative NSAIDs or surgical material induced AEP with unilateral consolidation appearing only on the operative side after VATS lobectomy for lung cancer surgery [9, 10].

According to the previous reports, various factors could potentially provoke AEP [6, 9, 10]. We used NSAIDs as postoperative pain medications for a few months starting just after the operation. Regarding whether NSAIDs could be the stimulant that provoked the AEP in this patient, the patient occasionally took NSAIDs for 1 month after complete remission of the AEP; however, his condition did not worsen. Considering this clinical course, we concluded that the NSAIDs unlikely triggered the AEP on this patient.

We also used a surgical material, polyglycolic acid sheet, during the operation to induce AEP; however, considering the whole clinical course and DLST result, meropenem administration was regarded to be the most crucial factor that provoked the AEP in this case.

We conclude that the combination of antibiotics and lung operative stimulation is the key factor that induced the unusual unilateral AEP pattern in the present case.

## Conclusion

Antibiotic therapy using meropenem for the treatment of postoperative bacterial pneumonia paradoxically caused unilateral eosinophilic drug-induced pneumonia worsening the patient’s condition. Drug-induced eosinophilic pneumonia usually occurs bilaterally. However, it is important to recognize that acute eosinophilic drug-induced pneumonia is a differential diagnosis of postoperative unilateral lung field infiltration, especially for postoperative patients of lung surgery because lung operation itself could be the trigger of this type of unilateral AEP.

## Data Availability

There is no available data and materials to be shared.

## Abbreviations

AEP: Acute eosinophilic pneumonia
BALF: Bronchoalveolar lavage fluid
CT: Computed tomography
DLST: Drug lymphocyte stimulation test
NSAIDs: Nonsteroidal anti-inflammatory drugs
VATS: Video-assisted thoracic surgery
